# Does the Tokyo guidelines predict the extent of gallbladder inflammation in patients with acute cholecystitis? A single center retrospective analysis

**DOI:** 10.1186/s12876-015-0365-4

**Published:** 2015-10-20

**Authors:** Peter C. Ambe, Hildegard Christ, Dirk Wassenberg

**Affiliations:** 1 Department of General, Visceral and Thoracic Surgery, St. Remigius Hospital Opladen, An St. Remigius 26, 51379 Leverkusen, Germany; 2 Department of Medical Statistics and Epidemiology, University of Cologne, Germany, Kerpener Str. 62, 50937 Köln, Germany; 3 Helios Klinikum Wuppertal, Department of Surgery II, Witten – Herdecke University, Heusner Str. 40, 42283 Wuppertal, Germany

**Keywords:** Acute cholecystitis, Laparoscopic cholecystectomy, Tokyo guidelines, Gallbladder inflammation, Gangrenous cholecystitis, Necrotizing cholecystitis

## Abstract

**Background:**

The Tokyo guidelines provide criteria for the diagnosis and classification of acute cholecystitis in three severity grades. However, no data exists on the predictive value of these guidelines. The aim of this study was to analyze the accuracy of the Tokyo guidelines as a predicting parameter for the severity of acute cholecystitis in patients undergoing laparoscopic cholecystectomy.

**Methods:**

A retrospective analysis of the charts of patients undergoing laparoscopic cholecystectomy for acute cholecystitis in a primary care hospital within a five-year period was performed. The preoperative severity grades were compared with the histological extent of inflammation.

**Results:**

One hundred thirty-eight patients; 79 with severity grade I, 33 with grade II and 26 with grade III were analyzed. The incidence of uncomplicated cholecystitis decreased with increasing severity grade, while the incidence of complicated cholecystitis increased with increasing severity. However, complicated cholecystitis was evident in an unexpectedly high number of cases with severity grade I. There was a significant correlation (*χ*^2^(1) = 10. 43, *p* = 0.01) between the preoperative severity grade and the extent of gallbladder inflammation on histopathology. Conversion to open surgery (14 vs. 5, *p* = 0.002) and complications (17 vs. 7, *p* = 0.001) were significantly higher in patients with preoperative severity grade II/III compared to patients with severity grade I.

**Conclusion:**

Worsening clinical severity correlated significantly with worseing pathology, findings from blood test and clinical outcomes; rates of conversion and morbidity. However, the Tokyo guidelines may have a tendency to underestimate the extent of inflammation in male patients with severity grade I and over estimate the difficulty of dissection in severity grade II.

## Background

Acute cholecystitis (AC) is frequently encountered in daily clinical practice. Since its publication in 2007 and the update in 2013, the Tokyo guidelines (TG 13 guidelines) for the diagnosis and management of acute cholangitis and acute cholecystitis rapidly gained popularity [1, 2]. These guidelines use clinical symptoms, findings from physical examination, blood test and imaging modalities to diagnose AC . Besides defining diagnostic criteria, the Tokyo guidelines also enable a classification of acute cholecystitis in three severity grades. Grade I describes a mild form of inflammation, grade II describes a moderate gallbladder inflammation, while grade III corresponds to severe gallbladder inflammation in association with organ failure [1–10].

Since the TG 13 grading system was developed to differentiate severity grades of inflammation, severe inflammation would be expected in grade II and III. Uncomplicated acute cholecystitis would therefore be expected in grade I, while complicated cholecystitis (gangrenous, necrotizing or empysematous AC) would be expected in grades II and III. Besides their diagnostic and severity grading properties, the TG 13 guidelines provide a severity dependent treatment algorithm for AC. Patients with grade I are generally candidates for laparoscopic cholecystectomy (LC). Patients with grade II could either undergo LC in centers with laparoscopic expertise or be managed via percutaneous cholecystostomy (PC). Patients presenting with grade III are generally managed with PC [8, 10].

In our department, as well as in many other centers in Germany, PC is rarely performed. Acute cholecystitis is generally managed via LC. Critically ill patients and those considered “not fit for anesthesia” are generally medically managed. After seeing a number of cases of extensive gallbladder inflammation in patients presenting with preoperative grade I cholecystitis, we questioned the accuracy of the TG 13 guideline in predicting the extent of gallbladder inflammation in patients with AC. The aim of this study therefore, was to investigate whether or not the preoperative disease severity grade per TG 13 correlates with the extent of gallbladder inflammation on histopathology.

## Methods

Following the approval of the ethics committee at the St. Remigius Hospital Opladen, Germany, data of patients managed with LC for acute cholecystitis within a five-year period (2009–2013) in the department of surgery was retrospectively reviewed. An informed consent was received from all patients for the use of their data in this study. Baseline data including age, sex, body mass index (BMI) and medical comorbidities as defined by the American Society of Anesthesiologists were retrieved for each patient.

Patients were admitted following presentation in the emergency department. The electronic charts were retrospectively reviewed and information on medical history, physical examination, ultrasound sonographic findings and blood chemistry at the time of presentation were extracted by two experienced surgeons. These data were employed to characterize disease severity as outlined in the Tokyo guidelines, Table 1.

**Table 1:** Diagnostic criteria and severity grading of acute cholecystitis as proposed in the TK 07/13 guidelines [1, 2]

Diagnostic criteria and severity grading of acute cholecystitis as proposed in the TK guidelines	
Diagnostic criteria	
- Local signs of inflammation: Murphy’s sign, pain/tenderness/mass in RUQ	
- Systemic signs of inflammation: fever, elevated CRP and WBC	
- Imaging : Ultrasound, CT, MRI	
Severity grading	
Grade I: mild acute cholecystitis	
- Acute cholecystitis in an otherwise healthy patient	
- ^a^mild inflammatory changes of the gallbladder, e.g., edematous cholecystitis	
Grade II: moderate acute cholecystitis	
- Clinical symptoms > 72 h	
- Palpable mass in the RUQ	
- Positive Murphy’s sign	
- WBC > 18.000 /ul	
- ^a^marked gallbladder inflammation e.g., gangrenous cholecystitis	
Grade III: severe acute cholecystitis	
- Acute cholecystitis with at least one of the following organ dysfunction	
- Cardiovascular: hypotension requiring catecholamine	
- Pulmonary	PaO_2_/FiO_2_ ratio < 300
- Renal	Creatinine > 2.0 mg/dl
- Neurologic	Decreased level of consciousness
- Hepatic	INR > 1.5
- Hematologic	Platelet count < 100.000/ul
- ^a^Severe gallbladder inflammation e.g., necrotizing cholecystitis	

*RUQ* right upper quadrant, *CRP* c - reactive protein, *WBC* white blood count, *CT* computed tomograhy, *MRI* magnet resonance imaging

^a^expected extent of gallbladder inflammation

As part of our departmental standards, all patients with AC are put on intravenous (i.v.) antibiotics, usually a combination of a cephalosporin and metronidazole. Although a policy of “same admission cholecystectomy” was maintained in our department, patients presenting with clinical symptoms > 72 h were managed medically and cholecystectomy was performed after 6 weeks. Equally, elective cases presenting after conservative treatment were managed after an interval of 6 weeks. Such patients were not included in this study. Patients who failed to recover following medical treatment were managed surgically, usually laparoscopically. These cases were excluded from analysis. Only cases managed with LC within 72 h of symptom onset (including cases of attempted LC) were included for analysis.

Laparoscopic cholecystectomy was performed in all cases using four incisions. The senior surgeon was one of four surgical attendings with experience in laparoscopic surgery. Since all patients with AC were placed on i.v. antibiotics upon admission, single shot antibiotic was administered depending on the interval between the last i.v. antibiotic application and the begin of surgery. Capnopneumoperitoneum was instilled via a sub-umbilical incision using a 12 mm trocar. A 10 mm trocar was placed at the epigastric position followed by two 5 mm trocars in the right upper quadrant. The maximum intraabdominal pressure was kept at 12 mmHg.

Laparoscopic gallbladder puncture was performed at the beginning of surgery as needed. The cystic duct and the cystic artery were managed using clips after which the gallbladder was dissected off the liver. Conversion to open surgery was performed due to the inability to clearly identify the structures within the triangle of calot.

Surgical documentation sheets and anesthesiology protocols were reviewed for information on the course of surgery. Complications and the length of hospital stay were retrieved from hospital discharge notes. Histopathology, which was performed in all cases, confirmed the diagnosis with a description of the extent of gallbladder inflammation using the following terminology: edematous, gangrenous or necrotizing. Since two pathologists signed the original pathology records, a reexamination of the slides was not performed. Edematous choleystitis was diagnosed in cases with gallbladder wall edema with interstitial fluid and dilated capillaries and lymphatics. Necrotizing cholecystitis was diagnosed following the presence of scattered superficial necrosis of the gallbladder wall. Gangrenous cholecystitis was characterized by loss of mucosal lining and vascular architecture with profuse inflammation changes secondary influx of inflammatory cells. The terminologies used to describe the extent of gallbladder inflammation on histopathology in this series are primarily german and correspond grossly with English terminologies used elsewhere [3]. Edematous cholecystitis was considered as “uncomplicated” while gangrenous and necrotizing cholecystitis were considered as “complicated”. The preoperative severity grades were correlated with the extent of inflammation on histopathology on one hand and clinical outcomes on the other hand.

The Statistical Package for Social Science (SPSS®), IBM, version 22 was employed to analyze the collected data. Continuous variables were described using absolute case numbers and percentages, central tendencies were described using means with the corresponding standard deviations. Statistical significances were calculated using the chi square test with the level of significance placed at *p* < 0.05. Furthermore, multiple comparisons using a one-way ANOVA were employed to test for significant differences amongst the different severity grades. Significant differences between the three severity grades were further investigated using the Bonferroni correction.

The primary endpoint was the correlation between preoperative severity grades and the extent of gallbladder inflammation on histopathology. Secondary endpoints included clinical outcomes: duration of surgery, rate of conversion, length of hospital stay (LOS) and rate of complication using the classification proposed by Clavien Dindo [11].

## Results

**Fig. 1:**
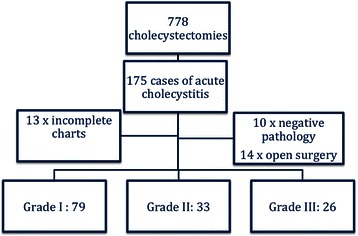
Distribution of the study population. Severity grading of the study population. In 14 cases AC was diagnosed following emergency laparotomy for acute abdomen

Within the period of investigation 778 cholecystectomies were performed in our department. Acute cholecystitis was the indication for surgery in 175 cases. After excluding cases with incomplete charts, negative pathology and primary open surgery, 138 cases of LC (including attempted LC) were included for analysis. Figure 1 shows the distribution of the study population, while the demographic features are summarized in Table 2.

**Table 2:** Summary of the baseline data of the study population

Demographic data of the study population					
Parameters		Number of cases/Severity grade			*P*-value
		Grade I	Grade II	Grade III	
Gender (F/M)		39/40	18/15	12/14	0.80
Mean age (yrs)		59.7 ± 14.5	67.7 ± 16.9	77.9 ± 9.2	0.31
Mean BMI (kg/m^2^)		26.9 ± 4.6	28.0 ± 5.1	29.7 ± 6.3	0.19
ASA	1	13	1	0	0.01
	2	46	15	5	
	3	20	16	17	
	4	0	1	4	

*F* female, *M* male, *yrs* years, *BMI* body mass index

A multiple one way ANOVA test showed that the mean values for WBC (F(2) = 31.844, *p* = 0.001) and CPR (F[1] = 26.091, *p* = 0.001) differed statistically significantly between the three severity groups. Post hoc tests using the Bonferroni correction revealed significantly lower mean CRP and WBC values in severity grade I compared to grades II and III, Table 3. Table 4 summerizes the criteria met in patients with severity grade II and III.

**Table 3:** Summary of the perioperative data in this study

Perioperative data of the study population				
Features	Grade I	Grade II	Grade III	*P*-values*
Mean WBC	11.8 ± 3.2 /μl	17.5 ± 5.7 /μl	17.9 ± 5.1 /μl	I vs. II : 0.001
				I vs. III: 0.001
				II vs. III: 0.91
Mean CRP	13.4 ± 9.4 mg/l	23.5 ± 11.6 mg/l	27.1 ± 9.2 mg/l	I vs. II : 0.001
				I vs. III: 0.001
				II vs. III: 0.25
Mean length of anesthesia	118.7 ± 26.2 min	130.1 ± 27.6 min	132.9 ± 31.4 min	I vs. II : 0.001
				I vs. III: 0.001
				II vs. III: 0.72
Mean duration of surgery	69.1 ± 22.0 min	77.4 ± 22.0 min	78.1 ± 26.3	I vs. II : 0.13
				I vs. III: 0.07
				II vs. III: 0.72
Mean length of postoperative stay	6 .0 ± 2.7 d	7.8 ± 3.3 d	10.4 ± 6.1 d	I vs. II : 0.07
				I vs. III: 0.001
				II vs. III: 0.02

*WBC* white blood count in cells / μl, *CRP* c-reactive protein in mg/l. *Bonferroni corrected *p*-values

*I* severity grade I, *II* severity grade II. *III* severity grade III

**Table 4:** Severity grading parameters for patients with grade II and III AC in this series

Severity grading of the study population	
Grade II (*n* = 33)	
WBC > 18.000/ul	16 cases
Palpable mass in RUQ	8 cases
Murphy’s sign	9 cases
Grade III (*n* = 26)	
acute renal failure	9 cases
INR > 2	10 cases
platelet count < 100.000/ul	6 cases
PaO2/FiO2 < 300	1 case

*WBC* white blood count, *RUQ* right upper quadrant, *INR*: international normalized ratio

**Fig. 2:**
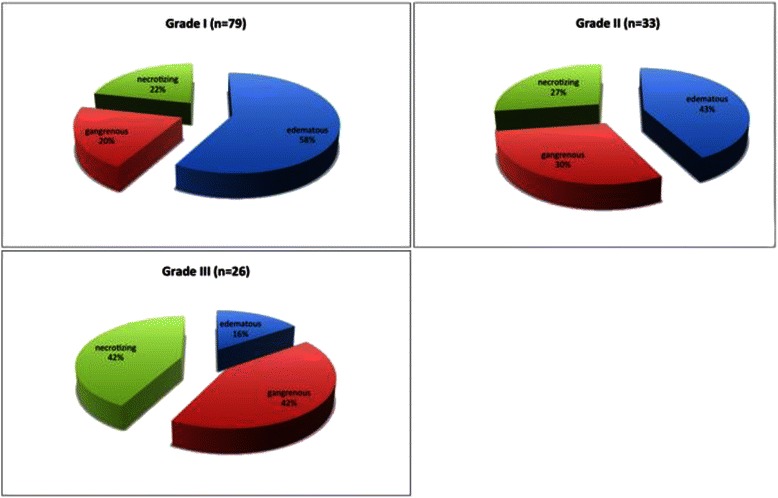
Histopathologic extent of gallbladder inflammation. The incidence of uncomplicated (edematous) cholecystitis decreased with increasing severity

The incidence of edematous cholecystitis was 58, 38 and 16 % for severity grade I, II and III respectively. Necrotizing cholecystitis was diagnosed in 22, 27 and 42 % of cases with severity grade I, II and III respectively, while gangrenous cholecystitis was recorded in 20, 30 and 40 % of cases with severity grade I, II and III. A Pearson’s chi square test demonstrated a significant correlation between the preoperative severity grade and the extent of gallbladder inflammation on histopathology, *χ*^2^(1) = 10. 43, *p* = 0.01. The histopathologic extent of gallbladder inflammation for each severity grade is presented in Fig. 2.

Complicated cholecystitis was found in 33 patients (33/79, 41.8 %) with severity grade I. There was no statistically significant association between the mean WBC (*p* = 0.204) and mean CRP (*p* = 0.374) in patients with and without complicated cholecystitis. Surgery lasted significantly longer in patients with complicated cholecystitis compared to those with uncomplicated cholecystitis, *p* = 0.004. Similar findings were computed for the rate of conversion (*p* = 0.023), the rate of morbidity (*p* = 0.027) and the need for ICU management (*p* = 0.016). However, there was no significant association between the extent of gallbladder inflammation and the LOS.

With the hope of identifying possible risk factors for extensive cholecystitis in patients with grade I AC, further analyses of the subgroup were performed. No statistically significant differences were found between patients with uncomplicated (edematous) cholecystitis and those with complicated (gangrenous and necrotizing) cholecystitis with regards to age (*p* = 0.456), BMI (*P* = 0.385), ASA score (*p* = 0.852), WBC (*p* = 0.769) and CRP (*p* = 0.421). Twenty-one of the 33 patients (63.6 %) with extensive (gangrenous or necrotizing) cholecystitis in the group with severity grade I were male patients. This difference was statistically significant (*p* = 0.050) compared to 19 male patients (19/ 46, 41.3 %) in the group with uncomplicated (edematous) cholecystitis.

Conversion from laparoscopic to open cholecystectomy (OC) was performed in 19 cases (13.7 %). The rate of conversion was 6.3 % (five cases) in the group with severity grade I. All five cases were diagnosed with complicated cholecystitis on histopathology. The rate of conversion was 15.2 % (five cases) in the group with grade II while nine cases (34.6 %) were converted in the group with grade III. There was no statistically significant difference between severity grade I and II (*p* = 0.136) as well as grade II and III (*p* = 0.081) with respect to the rate of conversion. However, significantly more conversions were performed in the group with severity grade III compared to grade I, *p* = 0.001.

Complications developed in 24 cases (17.4 %). Surgical complications were observed in 14 cases (10.1 %), while medical complications were seen in 10 cases (7.3 %). Seven complications (8.9 %) were recorded in the group with severity grade I. The rate of morbidity was 15.2 % (five complications) in the group with severity grade II and 46.2 % (12 complications) in the group with severity grade III. This difference was statistically significant (*p* = 0.01). Table 5 summarizes the complications recorded in this series.

**Table 5:** Postoperative complications classified by Dindo et al. [11]

Complications	Grade I	Grade II	Grade III
Grade I	0	1	3
Grade II	3	3	1
Grade III	4	1	2
Grade IV	0	0	6
Grade V	0	0	2^a^

Grade I: urinary tract infection 1x, liver abscess 1x, cystic duct leak 4x, 1x cholechocus stenosis

Grade II: wound infection 1x, pneumonia 3x (ICU management in 1x), urinary tract infection 1x

Grade III: surgical site infection 4x, wound dehiscence 1x, pneumonia 1x (ICU), cystic duct leak 2x, acute renal failure 2x (needing dialysis), ^a^mortality 2x (following pulmonary insufficiency and pulmonary embolism)

While stabilization in the intensive care unit was not necessary in patients with severity grade I, two patients (6.1 %) with severity grade II and 11 patients (42.3 %) with severity grade III were managed in the ICU following surgery. Two cases (1.4 %) of mortality (following pulmonary embolism and lung failure) were recorded in this series, both from the group with severe cholecystitis grade III (7.7 %).

The median length of postoperative hospital stay differed significantly between the three severity groups on multiple one-way ANOVA (F(2) = 14.126, *p* = 0.001). Post hoc tests using the Bonferroni correction revealed significant differences in the length of stay amongst the different severity grades, Table 3.

## Discussion

Laparoscopic cholecystectomy is a standard procedure for patients with benign gallbladder disorders including AC [12–14]. Cholecystectomy in severely ill patients however, has been shown to be associated with high rates of morbidity and mortality [13, 15, 16]. Equally, the extent of gallbladder inflammation has been shown to affect the outcome of LC [17, 18]. The Tokyo guidelines of 2007 and 2013 provide simple criteria not only to ease the diagnosis but also to enable a severity grading of acute cholecystitis and provide the most appropriate severity dependent treatment [1, 2]. However, no data exits on the correlation between the preoperative severity of AC and the extent of gallbladder inflammation on histopathology. The aim of this study was to investigate whether or not the preoperative severity grading of AC using the TG 13 guidelines could predict the extent of histopathologic gallbladder inflammation and the clinical outcomes in patients undergoing LC.

One hundred and thirty eight cases of LC, including attempted LC for AC were included for analysis. In our department AC is generally surgically managed. Critically ill patients and those not fit to undergo general anesthesia are managed medically and surgery is only performed following failure to recover under conservative treatment. PC therefore is not employed in our department, as is the case in many other centers in Germany. Therefore, the treatment algorithm proposed in the Tokyo guidelines with regard to PC for grade II and grade III AC were not employed in this study due to this regional difference.

The incidence of uncomplicated gallbladder inflammation decreased significantly with increasing severity grade. Similarly, the incidence of advanced gallbladder inflammation increased with increasing severity grade. Therefore, severe gallbladder inflammation in the form of gangrenous or necrotizing cholecystitis could be confirmed on histopathology in a significant majority of patients with preoperative severity grade II and III. Complicated cholecystitis was associated with longer duration of surgery, higher rate of conversion, higher rate of morbidity and increased need for ICU management. Thus worsening preoperative severity grade correlated significantly with worsening pathology. This finding is in accordance with the Tokyo guidelines [1, 2].

A rather intriguing finding in this study was the relatively high rate (over 40 %) of advanced cholecystitis in patients with grade I AC. Extensive cholecystitis (gangrenous and necrotizing cholecystitis) would normally be expected in patients with severity grade II/III and in patients with longstanding symptoms, that is > 72 h [17–19]. However, patients with symptoms > 72 h were not included in this series. One may argue that the timing of symptom onset can be an issue, especially since symptoms may be vague with varying intensity amongst individuals and sex. This finding clearly indicates that the TG 13 guidelines might underestimate the extent of gallbladder inflammation in patients with grade I AC and therefore questions the ability of the TG 13 guidelines in discriminating the extent of gallbladder inflammation in grade I.

Because of the potentially important clinical significance of the above mentioned finding in the group with severity grade I, subgroup analyses were proformed with the hope of identifying possible risk factors for an inaccurate classification of patients into severity grade I. Interesting, significantly more male pateints with severity grade I were diagnosed with complicated AC compared to those with uncomplicated AC. The male gender therefore seems to be a risk factor for extentive gallbladder inflammation in patients presenting with acute cholecystitis. A similar trend has been reported previously [20- 21].

Another interesting aspect of this study was the lack of statistically significant differences in the duration of surgery and the rate of conversion between patients with severity grades II and I. Both the duration of surgery and the rate of conversion have been used as to assess the surgical challenge in patients undergoing LC [22]. Conversion from laparoscopic to open cholecystectomy in this study was due to the inability to clearly identify the structures within the triangle of Calot. The lack of statistically significant difference in the duration of surgery amongst patients with grade I and grade II might argue that the difficulty of dissection associated with the surgical management of patients with grade II AC is over estimated by the Tokyo guidelines. This impression is further supported by the fact that there was no statistically significant difference in the rate of conversion amongst patients with grade I and grade II.

This finding however must be interpreted with caution due to the unexpectedly large number of cases with advanced cholecystitis in the group with grade I. A similar trend was observed between patients with grade II and grade III AC. Since experienced attending surgeons managed all patients included in this series, this trend cannot be blamed on surgical expertise. Equally, this trend cannot be blamed on “timing of surgery” since all cases were managed within 72 h of symptom onset.

The rate of morbidity increased with increasing disease severity. In fact, an alarming complication rate of 46.2 % was recorded in the group with grade III. Two cases (1.4 %) of mortality were recorded in this series following pulmonary failure and pulmonary embolism, both from the group with severity grade III (7.7 %). These results are unacceptable and definitely call for reflection. These findings might be interpreted as a justification of the delayed approach suggested by the Tokyo guidelines [1, 2].

Postoperative ICU management was needed in over 20 % of cases with severe gallbladder inflammation (grade II/III). In fact, 42.3 % of cases with grade III AC were admitted in the ICU following surgery. This was a rather unexpected finding since patients with severity grade III are expected to be candidates for ICU due to organ failure. However, only 11 patients with grade III AC (nine with renal failure, one with lung failure and one with very low platelet count (58.000 / ul)) required ICU management in this series. Since the threshold for ICU admission and the level of ICU treatment vary between institutes, one may argue that the indication for ICU admission was not liberally made in this series.

Taken together, our results showed that the preoperative severity of acute cholecystitis correlated significantly with the extent of gallbladder inflammation on histopathology. However, an unexpectedly high number of extensive gallbladder inflammation was seen in patients with grade I AC suggesting that the TG 13 guidelines might underestimate the extent of inflammation, especially in young and fit patients. The TG 13 severity grades correlated significantly with clinical parameters (WBC and CRP). Althought surgery lasted longer with increasing severity; there was no significant difference amongst the three severity grades with regard to the duration of surgery. However, rates of conversion and morbidity correlated significantly with disease severity. The predictive value of the TG 13 guidelines of the need for ICU management could not be justified in this series. This trend however must be interpreted with caution since the indications for ICU vary between institutions.

### Limitations

In our department, AC is generally managed with cholecystectomy. Thus alternative treatment options like percutaneous cholecystotomy (PC) were not used. Therefore, it remains speculative, if and how an alternative procedure like PC could have influenced the outcomes of patients with preoperative severity grade II/III. Furthermore, since the duration of symptoms > 72 h represents a major criterion in the severity grading per TG 13 guidelines, eliminating patients who presented with symptoms > 72 h might have resulted in underrepresentation of patients with severity grade II in this series. Furthermore, this study is limited by its retrospective design. Therefore results reported in this study must be validated in prospective studies.

## Conclusion

Worsening clinical severity correlated significantly with worseing pathology, findings from blood test and clinical outcomes; rates of conversion and morbidity. However, the Tokyo guidelines may have a tendency to underestimate the extent of inflammation in male patients with severity grade I and over estimate the difficulty of dissection in severity grade II. Therefore laparoscopic surgeons should be aware of this possible discripancy when managing patients with acute cholecystitis.

## Abbreviations

AC: Acute cholecystitis
ASA: American society of anesthesiologists
BMI: Body mass index
CRP: C- reactive protein
ICU: Intensive care unit
i.v.: Intravenous
LC: Laparoscopic cholecystectomy
LOS: Length of stay
OC: Open cholecystectomy
PC: Percutaneous cholecystotomy
SPSS: Statistical package for social sciences
TG 13: Tokyo guidelines 2013
WBC: White blood count
